# Inflammation in diabetes complications: molecular mechanisms and therapeutic interventions

**DOI:** 10.1002/mco2.516

**Published:** 2024-04-12

**Authors:** Lu Zhao, Haoran Hu, Lin Zhang, Zheting Liu, Yunchao Huang, Qian Liu, Liang Jin, Meifei Zhu, Ling Zhang

**Affiliations:** ^1^ Department of Biology and Medicine College of Life Science, Zhejiang Chinese Medical University Hangzhou China; ^2^ National Demonstration Center for Experimental Traditional Chinese Medicines Education (Zhejiang Chinese Medical University) College of Pharmaceutical Science, Zhejiang Chinese Medical University Hangzhou China; ^3^ Shanghai Key Laboratory of Compound Chinese Medicines, The Ministry of Education Key Laboratory for Standardization of Chinese Medicines, Institute of Chinese Materia Medica Shanghai University of Traditional Chinese Medicine Shanghai China; ^4^ Department of Critical Care Medicine The First Affiliated Hospital of Zhejiang Chinese Medical University (Zhejiang Provincial Hospital of Chinese Medicine) Hangzhou China

**Keywords:** diabetes complications, inflammation, molecular mechanisms, therapeutic interventions

## Abstract

At present, diabetes mellitus (DM) has been one of the most endangering healthy diseases. Current therapies contain controlling high blood sugar, reducing risk factors like obesity, hypertension, and so on; however, DM patients inevitably and eventually progress into different types of diabetes complications, resulting in poor quality of life. Unfortunately, the clear etiology and pathogenesis of diabetes complications have not been elucidated owing to intricate whole‐body systems. The immune system was responsible to regulate homeostasis by triggering or resolving inflammatory response, indicating it may be necessary to diabetes complications. In fact, previous studies have been shown inflammation plays multifunctional roles in the pathogenesis of diabetes complications and is attracting attention to be the meaningful therapeutic strategy. To this end, this review systematically concluded the current studies over the relationships of susceptible diabetes complications (e.g., diabetic cardiomyopathy, diabetic retinopathy, diabetic peripheral neuropathy, and diabetic nephropathy) and inflammation, ranging from immune cell response, cytokines interaction to pathomechanism of organ injury. Besides, we also summarized various therapeutic strategies to improve diabetes complications by target inflammation from special remedies to conventional lifestyle changes. This review will offer a panoramic insight into the mechanisms of diabetes complications from an inflammatory perspective and also discuss contemporary clinical interventions.

## Graphical abstract

Inflammatory response and its related signaling pathways in diabetes complications. In the pathogenesis of diabetes complications, inflammatory factors, inflammasomes, immune cells, adhesion molecules, chemokine, and chemokine receptor are involved in the progression of diabetes complications through a series of inflammatory reactions. Related signaling pathways, including NF‐κB, Toll‐like receptors, MAPK, JAK/STAT, PI3K/Akt, mediate a series of inflammatory responses of diabetes complications.

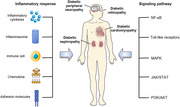

## 1 INTRODUCTION

Diabetes mellitus (DM), a metabolic disease characterized by hyperglycemia, can injury multiple organs and tissues. The international diabetes federation estimated the diabetic patients with age 20–79 years were over 536.6 million in 2021 and would rise to 783.2 million in 2045, as much as about 10% of adult population.^1^ Diabetes contains type 1 diabetes (T1DM) and type 2 diabetes (T2DM), and the former is a chronic autoimmune disease caused by innate insulin deficiency,^2^ while the latter is due to dysfunction of insulin secretion, thereby inflicting insulin resistance (IR).^3^ DM threatens the life span and quality to patients by directly or indirectly pathways. Owing to long‐term high glucose environment, both macrovessels and microvessels are impaired and will eventually inflict a series of complications, like diabetic cardiomyopathy (DCM), diabetic nephropathy (DN), diabetic retinopathy (DR), diabetic peripheral neuropathy (DPN), diabetic foot ulcer, and so on. Of note, most of patients with DM burden more than one complication at the early stage of DM, which indicates that there may be some related inducement between the complications.

It has been reported that plenty of factors were involved in diabetes complications including but not limited oxidative stress, metabolic disorders, immune inflammation, and so on.^4^ Among which, inflammation was deemed to play a pivotal role in the pathogenesis of diabetes complications. Slight inflammation was the main characteristic of DM, by mediating IR, vascular injury, and other related pathological processes.^5^ In addition, inflammation was used as a biomarker to predict the occurrences of diabetes complications including DCM,^6^ DN,^7^ DR,^8^ and DPN.^9^ Inflammatory cytokines such as tumor necrosis factor‐α (TNF‐α),^8^ transforming growth factor‐β (TGF‐β),^10^ interleukins (ILs),^11^ adhesion molecules,^12^, ^13^ and other genes related to macrophage could be used as biomarkers.^14^ Due to the complex roles of inflammation in the occurrence and development of diabetes complications, their relationship has not been fully clarified.

In this paper, we introduced the molecular mechanisms of inflammation and how it interfered diabetes complications, ranging from immune cell response, cytokines interaction to pathomechanism of organ injury. Furthermore, the representative drugs based on target inflammation to improve diabetes complications were discussed in the paper to support for follow‐up clinical research.

## 2 MOLECULAR MECHANISMS OF INFLAMMATION IN DIABETES COMPLICATIONS

Immune cells play critical roles in the inflammatory response. During the long‐term stimulation with high blood sugar, immune cells are activated and triggered a series of responses. For instance, when macrophages are activated, they initiate a series of downstream signaling pathways in the high glucose environment and release inflammatory cytokines. The TNF‐α, IL‐1β, IL‐6, or other inflammatory factors being released will activate various inflammatory signaling pathways, such as nuclear factor kappa‐B (NF‐κB), signal transducer, activator of transcription 3 (STAT3), and so on, resulting in organ damage.

### 2.1 Activation of immune cells

Monocytes and macrophages are important first line defenders in immune system of body. Once inflammation occurs, monocytes and macrophages will quickly gather to lesion area, and monocytes will differentiate into macrophage to destroy pathogens or cell fragments through phagocytosis and so on. Previous data showed the polarization of infiltrating macrophage was affected by the tissue microenvironment and external stimulation. For instance, macrophage could differentiate into M1 macrophages coming up abundant interferon‐γ (IFN‐γ) and TNF‐α microenvironment in situ.^15^ If some anti‐inflammatory cytokines such as IL‐4 and IL‐13 were present, M2 macrophage would be increased.^16^

**FIGURE 1:**
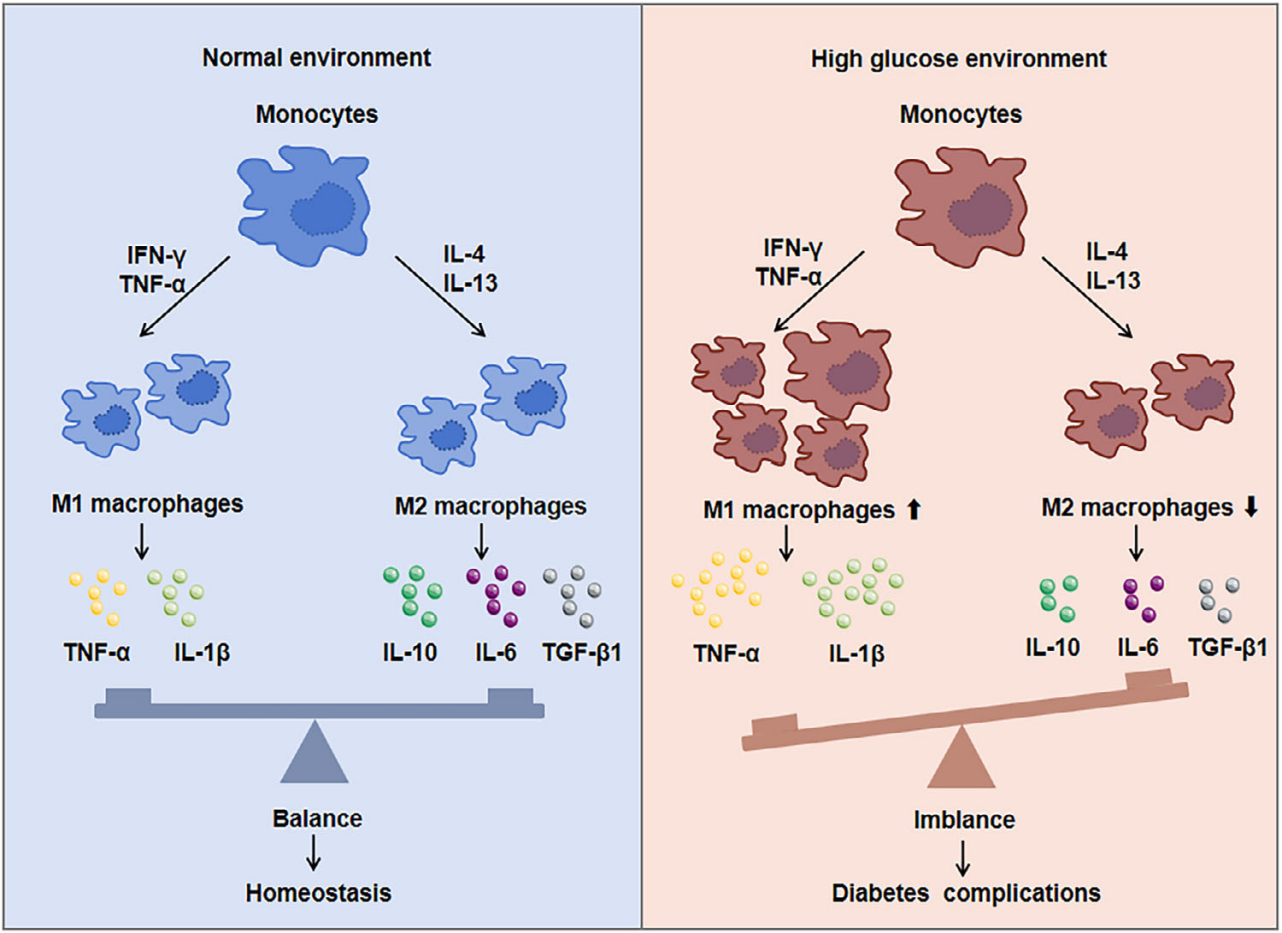
Activation of monocytes and macrophages in diabetes complications. Under normal environment, the ratio of M1/M2 macrophages is balanced, while the balance is disrupted under high glucose environment, the ratio of M1/M2 macrophages increases and produces a large number of proinflammatory cytokines, which aggravates the development of diabetic complications.

Studies have found that when diabetes complications occurred, monocytes were activated and recruited to the lesion area. Cytokines or chemokines secreted by monocytes also promoted much more monocytes recruitment and macrophage activation.^17^ Moreover, the ratio of M1/M2 macrophages increased and efferocytosis of macrophages gradually decreased to aggravate the DM (Figure 1). M1 macrophages secreted a large number of proinflammatory cytokines, resulting in IR, while M2 macrophages secreted anti‐inflammatory cytokines to enhance tissue repair and regeneration. For example, compared with healthy individuals, the number of infiltrating inflammatory monocytes and M1 macrophages in DCM patients were significantly increased.^18^ M1 macrophages secreted large amounts of proinflammatory cytokines, causing IR as well as accelerating the development of DCM.^19^ M2 macrophages secreted IL‐10 and slowed down the development of myocardial fibrosis,^20^ which could protect the heart in the early stages of DM. Therefore, transforming M1 macrophages into M2 phenotype and rebalancing the ratio of M1/M2 to inhibit myocardial fibrosis is a drug therapy strategy.^21^ As abundant immune cells infiltrated in patients with DN, the accumulation of macrophages indicated the renal dysfunction. Glucose promoted the transformation of M1 macrophages and podocytes apoptosis in DN rats and while activating M2 macrophages could protect podocytes from injury.^22^ On the one hand, proinflammatory M1 could change the integrity of podocytes. On the other hand, podocytes significantly promoted the migration of macrophages stimulated by high glucose medium.^23^ Also, macrophage‐depleted DM mice could reduce proteinuria and alter glomerular histology.^23^ Furthermore, the polarization of macrophages from M1 to M2 could reduce the inflammatory injury of kidney.^24^ At early stage of DR, both M1 and M2 microglia/macrophages were activated, but the numbers of M2 macrophages decreased with disease progress, and eventually resulted in retinal dysfunction. Previous data showed M2 macrophages‐related genes, such as *COL5A2* and *CALD1*, were highly expressed in immune cells in the retinal fibrovascular membrane and could be used as potential biomarkers for proliferative DR.^14^ It has been reported that asiatic acid reduced M1 polarization through TLR4/MyD88/NF‐κB p65 pathway and increased M2 polarization to prevent early DR.^25^ Histone demethylase Kdm6a could affect the gene transcription of macrophages, thereby affecting retinal thickness, visual acuity, and aggravating the development of DR.^26^ Macrophages can affect the neurovascular function of peripheral nerve tissue and regulate the occurrence of neuroinflammation. By analyzing the sciatic nerve of DPN mice, it was found that the markers of M1 macrophages such as TNF‐α and IL‐1β increased, and the markers of M2 macrophages such as IL‐10 and TGF‐β decreased,^27^ while after treatment, macrophages expressed lower levels of proinflammatory genes and higher levels of anti‐inflammatory genes.^27^ Therefore, interfering with macrophages to M2 polarization has positive effect to attenuate diabetes complications. In addition, the active macrophages could be regulated by T cell immunoglobulin domain and mucin domain‐3 (Tim‐3) and aggravated diabetic kidney damage, and the deletion of *Tim‐3* gene ameliorated podocyte injury and foot process disappearance in DN mice.^28^ Therefore, it can improve diabetes complications by reducing the recruitment of macrophages or affecting its activation and polarization in future.

### 2.2 Cytokines and chemokines interaction

As a small molecular protein with a wide range of biological activities, cytokines and chemokines can not only interact with each other but also participate in cellular pathological process, which eventually lead to diabetes complications.

#### 2.2.1 TNF‐α

TNF‐α expression was significantly increased in patients with DM, which could be used as a potential biomarker to predict the severity of the disease.^29^ In DCM, TNF‐α was overexpression in the heart and caused myocardial fibrosis, myocardial hypertrophy, and IR.^30^ TNF‐α was also as a biomarker for acute myocardial injury^29^ and enabled to increase itself expression as well as other inflammatory cytokines, which might lead to a vicious circle.^31^ Cardiovascular function would be improved in diabetic rats when TNF‐α expression was suppressed.^32^ Inhibition of TNF‐α resulted in suppression of P38 mitogen‐activated protein kinase (MAPK) and its downstream inflammatory mediators.^33^ PI3K/AKT signaling could be activated by TNF‐α as well, thereby inducing cardiomyocyte apoptosis and accelerating disease development.^34^ In DN, the level of TNF‐α in the urine was related to the presence and severity of microalbuminuria.^35^ TNF‐α/TIM‐3 deficiency macrophages could eliminate the podocyte injury.^28^ TNF‐α ablation of macrophages could improve various physiological indexes, such as albuminuria, plasma creatinine, renal macrophage recruitment, and so on.^36^ TNF‐α also enhanced the secretion of matrix metalloproteinases and promoted glomerular basement membrane deposition and fibrosis.^37^ In DR, TNF‐α increased the permeability of endothelial cells and in turn released adhesion molecules, while inhibiting TNF‐α expression could reverse the destruction of the blood–retinal barrier in DM mice and maintain the integrity of retinal tissue.^38^ Besides, its receptor TNFR1 could prevent retinal cell death induced by high glucose.^39^ Its receptor superfamily member CD40 was upregulated in the retina of diabetic mice, while CD40‐deficient mice affected nitration of retinal proteins and prevented retinal vascular degeneration.^40^ In DPN, TNF‐α expression was positively correlated with diabetic neuropathy symptoms and nerve conduction velocity, which was suggested that TNF‐α could catalyze DPN and be used as a biomarker of DPN.^41^, ^42^, ^43^ When TNF‐α was inhibited, the related symptoms of DPN could be improved, such as nerve conduction velocity increased, lamellar and axonal structure returning to normal, and so on.^44^ Downregulate TNF‐α expression and its receptors could also reduce the neuropathic pain of DM.^45^ Besides, TNF‐α stimulated IL‐6 production, in turn affecting the peripheral nerve axons and accelerating the development of DPN.^46^ Therefore, as an important proinflammatory cytokine, TNF‐α not only affected other cytokines or immune cells directly, but also mediated various pathological processes by affecting islet cells or IR.^47^

#### 2.2.2 Interleukin

ILs played an important role in diabetes complications. The level of IL‐6 was found to be increased in patients with DCM, which were involved in affecting myocardial fibrosis, cardiac hypertrophy, and glucose homeostasis during the development of DCM.^48^, ^49^, ^50^ When pyroptosis occurred, inflammatory substances such as IL‐1β and IL‐18 were released and involved in the pathogenesis of DCM.^51^ Accumulation of IL‐18 in the body could cause cardiomyocyte hypertrophy and myocardial fibrosis,^52^ and myocardial dysfunction was improved after using IL‐18 neutralizing antibodies.^53^ In DN, the serum IL‐18 and IL‐1β levels are positively correlated with DN stage. IL‐18 directly accelerated glomerular injury and its content was related to urinary albumin excretion rate.^54^, ^55^, ^56^, ^57^ IL‐6 was independently correlated with the risk of DN, overexpression of IL‐6 could induce podocyte apoptosis and growth arrest in high glucose environment,^11^ and it was also related to podocyte hypertrophy.^58^ IL‐17A was not only related to the decrease of glomerular filtration rate,^59^ but also mediated glomerular injury and interstitial fibrosis by mediating autophagy.^60^ In DR, IL‐6 participated in cell recruitment of microglia and the integrity of blood–retinal barrier.^61^ IL‐17A could induce retinal neurons and retinal endothelial cells apoptosis, activate retina Müller cells and disrupt its function, and deform the capillaries.^62^, ^63^, ^64^ IL‐8 was also considered to be the risk of proliferative DR.^65^ The polymorphism of IL‐10 gene was associated with the occurrence and development of DR.^66^ IL‐1β and IL‐18 mediated the scorched death of retinal cells, pericyte apoptosis, endothelial cell permeability, and retinal angiogenesis.^67^, ^68^ In DPN, IL‐6 could improve the neurovascular function, promote nerve regeneration, and protect Schwann cells injury from high glucose.^69^, ^70^, ^71^, ^72^ IL‐1β expression was increased in DPN rats and the ultrastructure of myelin and the axon of DPN rats treated with IL‐1β receptor antagonist got more highly protective effect than normal rats.^73^ Therefore, ILs can be used as a biomarker for the prevention and detection of diabetes complications.

#### 2.2.3 TGF‐β1

TGF‐β1 was one of the strongest cytokines to induce fibrosis in organisms.^74^ TGF‐β1 could activate its downstream protein Smads to induce myocardial fibrosis,^75^ renal fibrosis,^76^ and retinal fibrosis.^10^ In DCM, TGF‐β1 could promote myocardial fibrosis as well as induce DCM progress through TGF‐β1/Smads pathway.^75^ The decreased content of TGF‐β1, Smad2, and Smad3 protein improved heart function and prevented DCM devolopment.^77^ Smad3 deficiency had been reported to inhibit myocardial fibrosis and cardiac inflammation in db/db mice.^78^ In DN, TGF‐β1 contributed to the repair process of renal injury and was a key regulator of renal inflammation.^79^ It could exert its anti‐inflammatory effect by inhibiting glomerular cell mitosis and cytokine response^80^ and mediate the transformation of renal tubular epithelial cells into myofibroblasts to induce renal fibrosis through epithelial–mesenchymal transition (EMT). Specific Smad3 inhibition could improve the progression of disease by inhibiting TGF‐β1/Smads signal.^81^ Moreover, TGF‐β1 could decrease nephrin and lead to albumin permeability, suggesting that TGF‐β1 played a crucial role in hyperalbuminuria production.^82^ Studies have proved that TGF‐β1 was not only a biomarker and pharmacological target for DR,^10^ but also an inhibitory factor for retinal neovascularization in proliferative retinopathy.^83^ TGF‐β1 could affect vascular maturation and endothelial cell proliferation.^84^ Previous study has been shown that TGF‐β1 at low concentration promoted endothelial cell proliferation and migration, while inhibited these effects at high concentration.^85^ In addition, TGF‐β1 was involved in retinal fibrosis and its activation played an additive role in promoting the overexpression of extracellular matrix proteins in Muller cell.^86^ In DPN, there was a positive correlation between TGF‐β1 and nerve conduction velocity, suggesting that it might be used as a biomarker of DPN.^9^ Also, inhibition of TGF‐β1 could inhibit neuronal apoptosis and regulate extracellular matrix and neuronal demyelination.^87^, ^88^ Therefore, TGF‐β can be used as a therapeutic target for complications of diabetes.

#### 2.2.4 Chemokines

Chemokines can be roughly divided into four subfamilies CC, C, CXC, and CX3C families. Monocyte chemotactic protein‐1 (MCP‐1) protein was extensive expressed in myocardial tissue and involved in the pathogenesis of DCM^89^; it activated mononuclear macrophages to enhance the inflammatory response and finally promote fibrous tissue deposition.^90^ Downregulation of MCP‐1 levels with drugs treatment slowed the development of DCM.^91^ Inhibition CCR5 could reduce the proportion of M1 macrophages in rat heart tissue as well as block M2 macrophages due to nuclear receptor subfamily 4 group A member 2 induction in vitro.^92^ CXCR4 antagonists could reduce diabetes‐induced cardiac fibrosis.^93^ CCR2 was upregulated in diabetic heart; knockdown of CCR2 could reverse cardiac fibrosis, improve cardiac function, and reduce M1 macrophage infiltration.^94^ CCL2 and its receptor CCR2 could mediate VCAM‐1 to injury glomerular endothelial cells in DN.^95^ CXCL9 was found to be increased in serum and urine of DN patients; it regulated the numbers of podocytes by Janus kinase‐signal transducer and activators of transcription pathway (JAK/STST3) pathway.^96^ CXCR4 had a protective effect on the kidney to promote renal tubular cell survival but it could be affected by ligand‐inactivation gendopeptidases.^97^ CXCL10 was involved in occurrence of renal fibrosis after high glucose stimulating in DN and restoring the abundance of CXCL10 could reduce the occurrence of fibrosis.^98^ CXCL13 might play a role in the recruitment of T‐follicular helper (Tfh) cells in different stages of DR.^99^ CXCL1 could change the blood–optic retina barrier of DR through neutrophil recruitment, thus becoming a potential new therapeutic target.^100^ CCR2/CCR5 inhibitors could reduce the content of stromal cell‐derived factor‐1 (SDF‐1), intercellular cell adhesion molecule‐1 (ICAM‐1) and retinal vascular permeability in DM animals.^101^ CCR2 knockout microglia inhibited TNF‐α expression in retinal neurons, significantly.^102^ CXCL2 regulated sciatic nerve and schwann cells apoptosis through NOD‐like receptor thermal protein domain‐associated protein 3 (NLRP3) pathway.^103^ SDF‐1 and its receptor CXCR4 mediate calcium influx and excitability of dorsal root neurons, and using its inhibitors could relieve neuropathic pain.^104^ CXCL12/CXCR4 and CCR4 were also found to be one of the targets for the treatment of diabetic neuropathic pain.^105^, ^106^ CXCL1, CXCL5, CXCL9, CXCL10, CXCL11, and CXCL12 were important in nociceptive transmission and promoted the recruitment and penetration of CD8+ T cells in DPN.^107^, ^108^ Therefore, chemokines could attract and activate inflammatory cells to move toward the inflammatory site, participate in the regulation of inflammatory response, and be involved in tissue injury repair and the occurrence of vascular lesions in DM.

Taken together, immune cell activation and cytokines interaction were necessary in the progress of diabetes complications, which deserved to further explore the specific role of macrophage subsets and studied how specific subsets play a role in diabetes complications. At the same time, the relationship between macrophages and other cells, such as endothelial cells and fibrous cells, should be attended. Furthermore, exploring efficient inhibitors of cytokines provide valuable insights into understanding their transmission within cells. This could potentially lead to discovering novel targets for treating diabetic complications.

## 3 IR AND INFLAMMATION

As one of the pathological factors of diabetes, IR was the cell abnormal response to insulin stimulation, which caused the body to produce much more insulin, compensatively.^109^ Previous studies showed that inflammation was closely related to IR; the relevant inflammatory signaling pathways and inflammatory mediators were worth exploring.^110^

### 3.1 Impaired insulin signaling pathways

Several studies have shown that activation of phosphatidylinositol 3‐kinase/protein kinase B (PI3K/AKT) and MAPK signaling pathways could promote the occurrence of hyperglycemia and IR.^111^, ^112^ C‐Jun N‐terminal kinase (JNK),^113^ JAK/STAT,^114^ and NF‐κB^115^ were closely related to IR. The activity of JNK and insulin receptor substrate 1 (IRS‐1) serine phosphorylation inhibited the occurrence of IR.^116^ JAK/STAT could affect the secretion of proinflammatory cytokines, such as TNF‐α and IL‐6, thus mediating IR.^114^, ^117^ IKK/NF‐κB signal pathway was involved in IR in DM.^115^, ^118^ In DCM, activating PI3K/AKT signal pathway or inhibiting NF‐κB signal pathway could reduce the occurrence of IR,^119^, ^120^ and AKT2 deficiency resulted in severe glucose intolerance, myocardial contractile dysfunction, cardiomyocyte apoptosis, and impaired cardiac function.^121^, ^122^ IRS1/PI3K/AKT pathway was the main signal regulation pathway of podocyte IR, and blocking this pathway would induce EMT and glomerulosclerosis in DN podocytes.^123^ Further study found that AKT2 deficiency induced obvious podocyte damage including foot process fusion and podocyte apoptosis.^124^ In addition, inhibition of adenosine 5′‐monophosphate (AMP)‐activated protein kinase α (AMPKα) activity could result in podocyte damage and albuminuria.^125^ In DR, mammalian target of rapamycin (mTOR) activation could reverse the occurrence of IR in retinal pigment epithelial cells.^126^ JNK/S6K1‐induced IR was linked with retina injury as well.^127^ In DPN, diabetic neuropathy was affected by previous IR even though blood glucose was controlled at normal levels.^128^ Activated JNK could induce IRS‐1 serine phosphorylation, inhibit AKT and GSK3β serine phosphorylation, and promote the occurrence of IR in diabetic neuropathy.^129^ In addition, increase of serine phosphorylation of IRS2 changed insulin support in neurons and promotes peripheral nerve dysfunction.^130^ Therefore, the new strategy to attenuate IR would have positive significance for the improvement of diabetes therapy.

### 3.2 Inflammatory mediators disrupting glucose homeostasis

**FIGURE 2:**
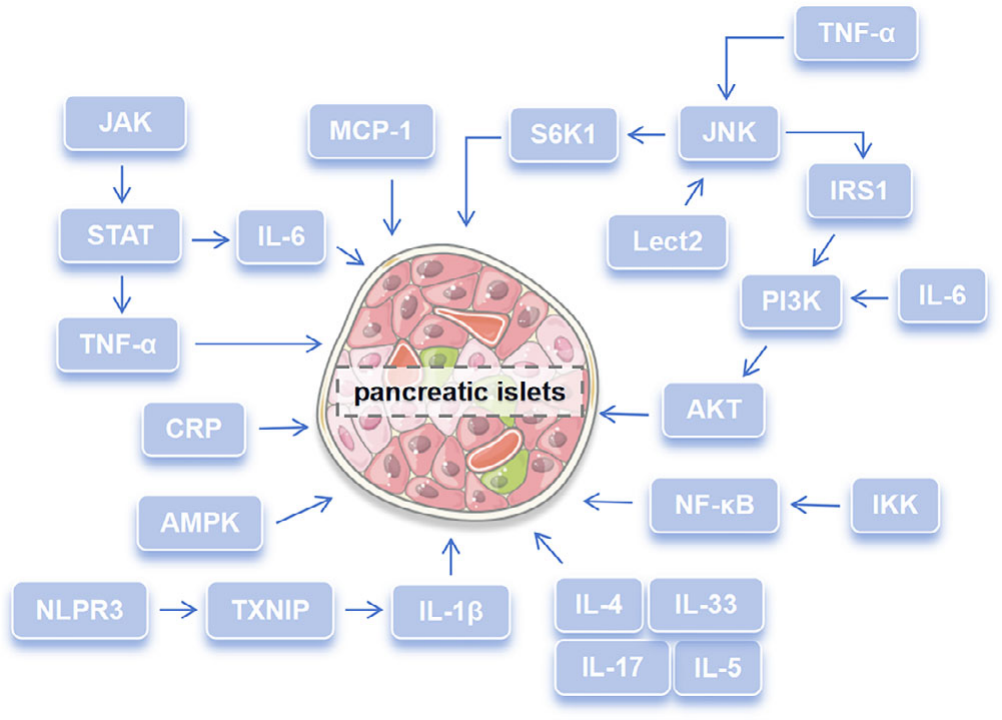
Signaling pathways and inflammatory mediators are involved in insulin resistance. Plenty of signaling pathways such as IKK/NF‐κB, IRS1/PI3K/AKT, JNK/S6K1, AMPK, JAK/STAT, and so on can mediate insulin resistance, while related inflammatory mediators including TNF‐α, IL‐4, IL‐5, IL‐33, IL‐17, IL‐6, IL‐1β, CRP, Lect2, NLRP3 inflammasome, and so on affect the occurrence of insulin resistance.

The abnormal level of various inflammatory mediators caused intracellular inflammatory response and blocked insulin signal transmission. As one of the most important proinflammatory mediators, TNF‐α expression increased at the early inflammatory stage and insulin signal transduction was damaged and leaded to IR by IRS‐1 and JNK1/2.^131^, ^132^ In high glucose environment, the levels of IL‐6 and IL‐1β were significantly increased, which were closely related to IR.^117^, ^133^ Among them, IL‐1β could not only inhibit insulin signal transduction in macrophages to cause abnormal insulin secretion,^133^ but also attracted proinflammatory cells to migrate to islets and damage it.^134^ IL‐6 could trigger the JAK/STAT and PI3K signal pathway to induce IR.^135^ Moreover, IL‐6 could improve glucose tolerance, reduce gluconeogenesis genes expression in the liver, and increase the phosphorylation of AKT, thus maintaining insulin homeostasis.^136^ IL‐17‐deficient mice showed higher glucose tolerance and insulin sensitivity, which was suggested that IL‐17 was a negative regulator of glucose metabolism.^137^ IL‐33 produced by islet mesenchymal cells could promote β‐cell function through islet‐resident group 2 innate lymphoid cells, and they interacted with each other to promote insulin secretion.^138^ On the contrary, some anti‐inflammatory cytokines, such as IL‐4 and IL‐5, could increase insulin sensitivity and slow down the occurrence of IR.^139^ Leukocyte cell‐derived chemotaxin 2 (LECT 2), as a hepatocyte factor, caused IR in skeletal muscle through JNK signal pathway.^140^ The MCP‐1 increased in adipose tissue contributed to macrophage infiltration and induced IR.^141^ NLRP3 inflammasome cooperating with ROS‐liberated TXNIP derived pancreatic islets to secrete IL‐1β and delayed pancreatic islets function damage in the response of chronic elevated glucose.^142^ C‐reaction protein (CRP) could directly regulate the central role of leptin and hypothalamic signal to affect insulin sensitivity and glucose homeostasis.^143^ Enhanced IκB kinase beta activity could improve insulin sensitivity and consequently had a beneficial role in glucose homeostasis.^144^ Therefore, inflammatory mediators may affect the homeostasis of glucose and mediate the occurrence and development of diabetes by inhibiting insulin secretion and affecting insulin sensitivity. Signaling pathways and inflammatory mediators involved in diabetes complications were summarized in Figure 2 and new strategy targeting IR would have positive significance for the improvement of diabetes therapy.

Taken together, inflammatory mediators and various inflammatory signal pathways interact with each other, in turn affecting glucose homeostasis and mediating the complications of diabetes. In the future, it can deeply study the gene regulation mechanism of inflammatory mediators, including the role of transcription factors, DNA methylation, and so on. At the same time, it can also analyze different types of cells to further reveal the similarities and differences of the regulation mechanism of inflammatory mediators on glucose homeostasis in different cells. Further it is essential to understand the relationship between cytokines and insulin signaling pathway, which can provide new ideas for the treatment of DM in future.

## 4 INFLAMMATION‐MEDIATED COMPLICATIONS IN DIABETES

According to the different pathological mechanism, diabetes complications could be divided into microvascular complications and macrovascular complications. The former includes DN, DR, and DPN, while the latter includes cardiovascular disease, cerebrovascular disease, peripheral arterial disease, and so on.^145^ In patients with DM, long‐term hyperglycemia activated a variety of inflammatory signal pathways and inflammatory mediators, resulted in aggravating the development of diabetes complications, and eventually led to damage of various organs.^146^ Inflammation could participate in the many pathological processes such as apoptosis, vascular endothelial damage, plaque formation, tissue fibrosis, and so on.^147^ The common complications in DM patients, with the purpose of clarifying the relationship between inflammation and cardiovascular disease, nephropathy, retinopathy, and neuropathy, are discussed as follow.

### 4.1 Cardiovascular system

When the body is subjected to a prolonged high‐glucose environment, it can result in vascular damage, which in turn can lead to a variety of cardiovascular conditions, including DCM, coronary heart disease, and atherosclerosis (AS). Individuals with DM have a significantly higher incidence of these cardiovascular diseases compared with those without diabetes.

#### 4.1.1 AS and inflammation

AS was caused by vascular endothelial injury that led to cholesterol and platelets deposited in the blood vessel wall and formed plaques, then finally resulted in vascular obstruction or stenosis. Under long‐term stimulation of inflammation in a high glucose environment, arterial endothelial cells were dysfunctional and released adhesion molecules, resulting in adhesion between white blood cells and small blood vessels. Meantime, because of increase of vascular endothelial permeability, low‐density lipoprotein (LDL) was accumulated and was oxidized and influenced monocytes.^148^ Oxidized LDL could induce macrophages to express scavenger CD36 and make self‐antigen presentation disappear, then transforming into foam cells. With the accumulation of these foam cells and other inflammatory cells, AS plaques were eventually formed. In addition, while the inflammatory response persisted, it would induce various inflammatory cytokines expression, stimulate macrophages activation, trigger a series of cascade reactions, and accelerate AS.^149^ Therefore, AS was closely related to the activation of macrophage and the secretion of adhesion molecules or inflammatory cytokines. As one of the common triggered of DM and AS, the dynamic changes of inflammation level were closely related to the occurrence of AS in patients with diabetes.^150^ Intervention of inflammatory levels in patients with DM could delay the occurrence of AS.^151^

#### 4.1.2 Cytokines impact on heart function

**FIGURE 3:**
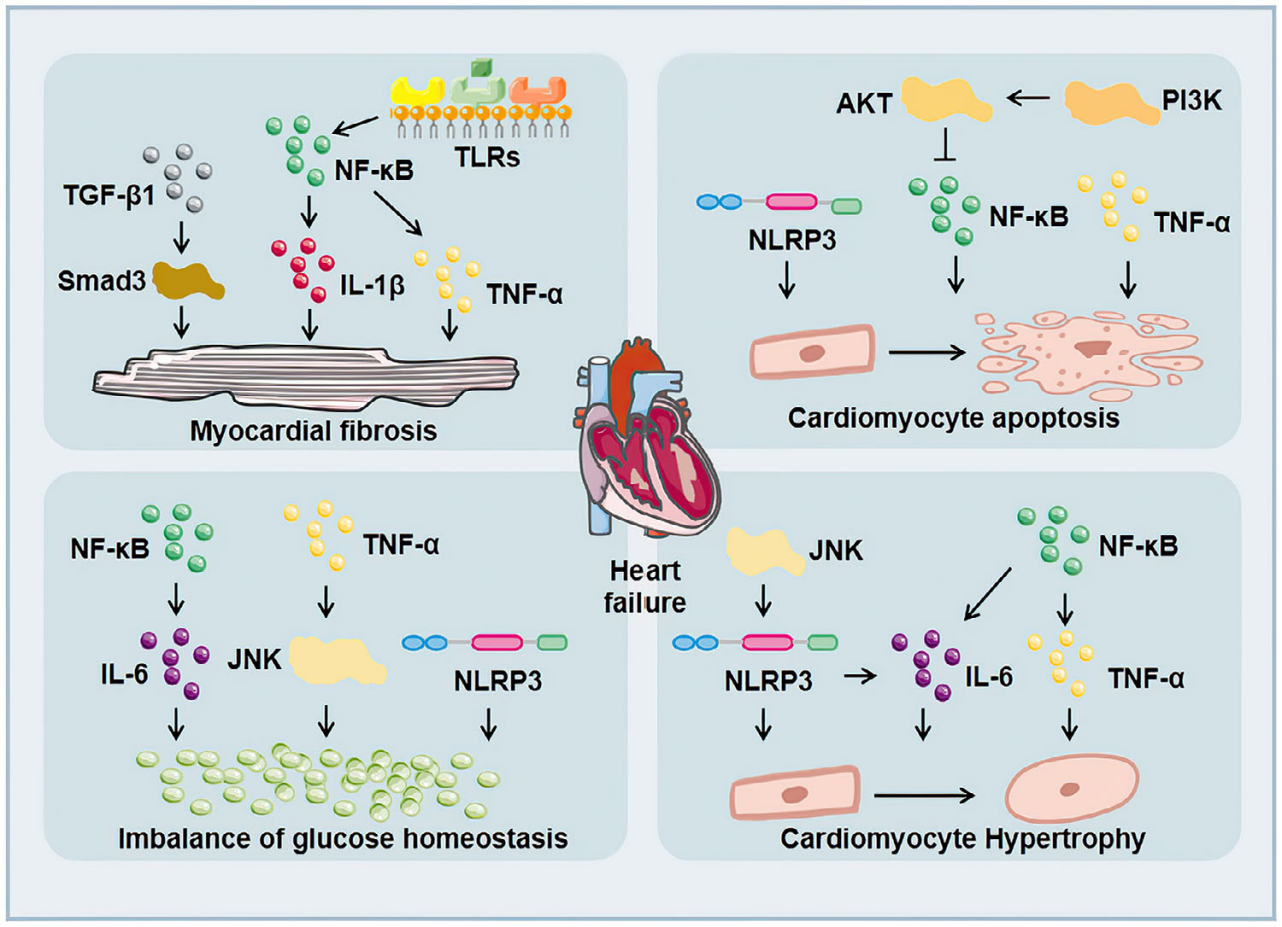
Cytokines that regulate cardiac pathological processes. TNF‐α causes cardiomyocyte apoptosis, cardiomyocyte hypertrophy, myocardial fibrosis, and impaired glucose homeostasis. IL‐6 promotes cardiomyocyte hypertrophy and impaired glucose homeostasis. TGF‐β1 participates in myocardial fibrosis. NLRP3 inflammasome engages in the process of glucose homeostasis, cell apoptosis, cardiomyocyte hypertrophy and impaired glucose homeostasis.

Under the influence of long‐term chronic hyperglycemia, the heart of DM patients was seriously damaged, resulting in myocardial hypertrophy, myocardial fibrosis, myocardial ischemia, myocardial infarction, and might eventually lead to heart failure, which was threaten to the health of DM patients. The incidence rate of heart failure in DM patients was two to four times higher than that in non‐DM patients.^152^ Cytokines and inflammasomes accelerated the injury of cardiac function (Figure 3). TNF‐α in the heart caused cell apoptosis, myocardial hypertrophy, and myocardial fibrosis.^30^ IL‐6 promoted cardiac hypertrophy, proliferation of cardiac fibroblasts, and collagen production in diabetic rats.^48^, ^49^ TGF‐β1 activated its target protein Smads and promoted myocardial fibrosis in DCM.^75^ NLRP3 inflammasome activation participated in the process of glucose homeostasis,^142^ cardiomyocyte apoptosis,^153^ cardiomyocyte hypertrophy,^154^ and myocardial fibrosis.^155^ ICAM‐1 was related to Ang II‐induced cardiac remodeling.^156^ Related signal pathways also affected inflammatory cytokines expression, thus affecting the function of the heart. For example, Toll‐like receptors (TLRs) might accelerate many inflammatory cytokines expression, such as IL‐6, IL‐1β, TGF‐β, and so on.^157^ Upregulation of TLR4 could activate NF‐κB in cardiomyocytes and promote the production of inflammatory cytokines, such as TNF‐α and IL‐1β, causing myocardial inflammation and myocardial fibrosis in DCM.^158^, ^159^ *TLR* gene knockout could reduce the level of NF‐κB phosphorylation, adhesion molecules, and proinflammatory cytokines.^160^, ^161^ NF‐κB affected the release of related inflammatory cytokines, such as TNF‐α, IL‐6, and IL‐1β, which mediated cardiac hypertrophy, myocardial fibrosis, cardiomyocyte apoptosis, and so on.^162^, ^163^ The P38 pathway could regulate a variety of genes including TNF‐α and TGF‐β. At the same time, its activity could also be enhanced by proinflammatory cytokines such as TNF‐α and IL‐6, thereby exacerbating the inflammatory response.^164^ JNK was a key upstream molecule of NF‐κB^165^ and it could be activated by inflammatory cytokines such as TNF‐α, which induced IR.^166^ TGF‐β1/JNK was related to myocardial fibrosis in DCM.^167^ In addition, JNK was involved in regulating NLRP3 inflammasome activation in macrophages.^168^ IL‐1β, IL‐6, and TNF‐α could stimulate JAK/STAT after binding to the receptor, thereby initiating the expression of downstream inflammation‐related target genes.^169^ The activation of JAK/STAT further stimulated cytokines, aggravated the inflammatory response,^170^ and participated in myocardial fibrosis.^171^ Besides, activation of the PI3K/AKT pathway could also inhibit NF‐κB expression to alleviate high glucose‐induced cardiomyocytes damage.^172^ Therefore, inflammation accelerates the damage of cardiac structure and function in patients with DM, and eventually lead to heart failure.

### 4.2 Nephropathy

DN is the renal damage caused by diabetic microangiopathy, characterized by persistent proteinuria. It was the most common chronic kidney disease at present, more than 50% of DM patients would develop DN.^173^ Inflammation participates in variety of pathological process of DN including renal tubular fibrosis, inflammatory cell infiltration, extracellular matrix accumulation, podocyte autophagy, and so on.

#### 4.2.1 Inflammation damage to renal structures

**FIGURE 4:**
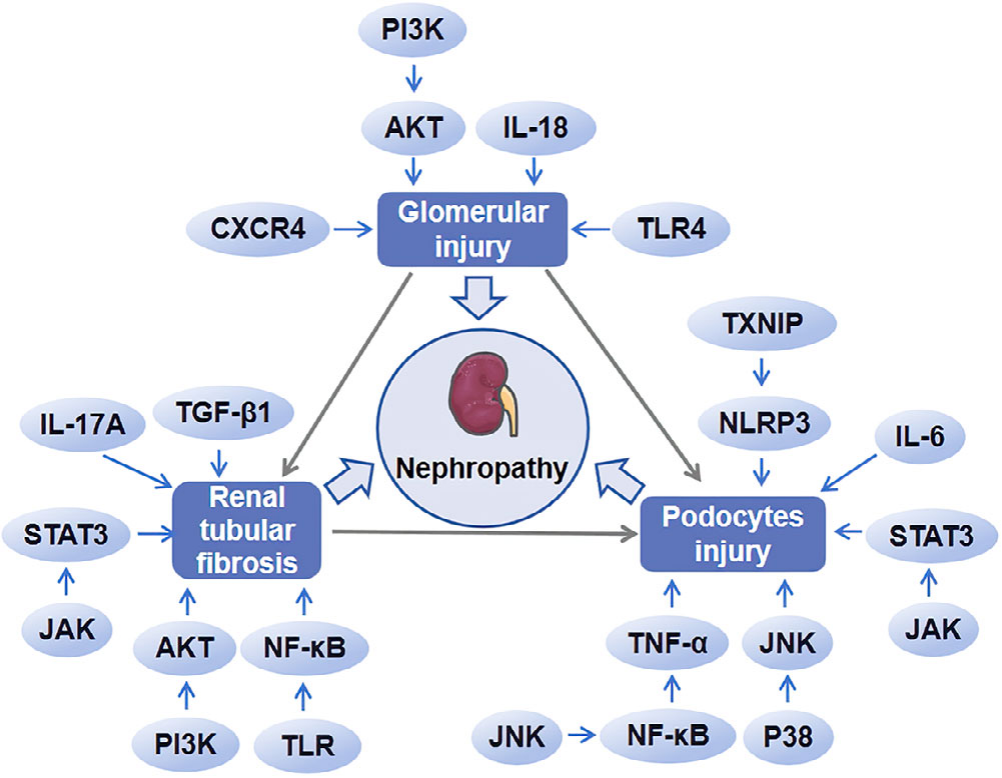
Inflammation damage to the kidneys. Multiple inflammatory reactions can play a role in aggravating the development of DN. PI3K/AKT, IL‐18, CXCR4, and TLR4 aggravate glomerular injury. TXNIP/NLRP3, JAK/STAT3, P38/JNK, TNF‐α, and IL‐6 participate in podocytes apoptosis. PI3K/AKT, TLR/NF‐κB, JAK/STAT3, TGF‐β1, and IL‐17A influence renal tubular fibrosis.

In the pathological process of DN, the aggravation of inflammation damage the structure of the kidney, thus leading to the deterioration of the disease. In the early stage of DN, tubular injury, glomerular hypertrophy, and glomerular basement membrane thickening occur by the effect of inflammatory cytokines and immune cells. Glomerulosclerosis, tubulointerstitial fibrosis, and other symptoms emerged at the late stage of DN.^174^ The glomerular filtration membrane consisted of endothelial cells, glomerular basement membrane, and podocytes from the inside to the outside. TNF‐α and IL‐6 reduced the capillary space between endothelial cells, resulting in impaired vasodilation function. At the same time, inflammation increased the adhesion of leukocytes to endothelial cells and promoted the folding of red blood cells, which reduced NO production and promoted the occurrence of EMT, and then finally led to renal fibrosis.^175^ In a high glucose environment, accompanied by thickening of the glomerular basement membrane, podocytes appeared hypertrophy, EMT, apoptosis, and exfoliation, which interfered with the normal structure and function of glomeruli, ultimately leading to abnormal glomerular filtration and the occurrence of proteinuria.^176^ A variety of inflammatory reactions could affect the structure and function of kidney. TNF‐α could mediate podocyte injury^28^ and affect various physiological indexes, such as albuminuria, plasma creatinine, and renal macrophage recruitment.^36^ IL‐18 could directly accelerate glomerular injury^56^ and IL‐6 could induce podocyte apoptosis and growth arrest in high glucose environment.^11^ IL‐6 also played a role in podocyte hypertrophy induced by high glucose through JAK2/STAT3 pathway.^58^ IL‐17A could affect the expression of CD40 and TGF‐β1^59^ and participate in the process of podocyte injury and renal interstitial fibrosis.^60^ TGF‐β1 could mediate the transformation of renal tubular epithelial cells into myofibroblasts to induce renal fibrosis through EMT.^76^ NLRP3 silencing could improve podocyte autophagy, reduce podocyte injury, and inhibit renal fibrosis.^177^, ^178^ Inhibition of TXNIP/NLRP3 pathway could inhibit apoptosis.^179^ CXCR4 could mediate the death of renal tubular epithelial cells^97^ and CXCL9 was related to podocyte injury.^96^ Inhibiting the activation of NF‐κB pathway could reduce renal inflammation and improve renal fibrosis.^180^ APMK/Sirt1/NF‐κB pathway could affect inflammation in DN,^181^ and NF‐κB/TNF‐α pathway mediated podocyte injury in DM rats.^28^ The lack of *TLR4* gene or inhibition of TLR4 expression improved urinary protein, glomerular hypertrophy, and renal tubular injury.^182^, ^183^ Also, the activation of TLR under high glucose could activate NF‐κB and the subsequent inflammatory and fibrotic reactions.^183^ JNK inhibitor reduced the activity of NF‐κB, adhesion molecules expression, and the infiltration of inflammatory cells in DN mice.^184^ The activation of P38/JNK signal pathway could promote podocyte apoptosis and aggravate renal injury in DN mice.^185^ JAK/STAT3 participated in podocyte damage,^96^ renal interstitial fibrosis,^186^ and podocyte hypertrophy.^58^ PI3K/AKT signal was involved in renal tubulointerstitial fibrosis, tubulointerstitial cell injury, and glomerulosclerosis.^123^, ^187^ Therefore, various inflammatory reactions will aggravate the damage of renal structure in DN patients and eventually lead to renal dysfunction (Figure 4).

#### 4.2.2 Altered filtration and kidney function

The decrease of proteinuria and glomerular filtration was a typical manifestation of DN, and it could be influenced by inflammation. Under the condition of high glucose, inflammatory cells such as macrophages infiltrated the glomeruli and released related inflammatory cytokines.^188^ Inflammatory cytokines such as IL‐6 and TNF‐α caused extracellular matrix proliferation, glomerular fibrosis, filtration barrier destruction, and albuminuria.^189^ During the process of DN, mesangial cell hypertrophy, podocyte rearrangement, and podocyte decrease appeared in glomeruli, changing the permeability of glomerular capillaries, and eventually led to glomerular filtration barrier damage and albuminuria.^190^ Podocytes are an important part of glomerular filtration barrier; its related inflammatory signaling pathways, such as NF‐κB, TLRs, JAK/STAT and PI3K/AKT, mediate glomerular filtration of podocytes. NF‐κB inhibitor reversed HG‐treated macrophage‐mediated podocyte injury.^28^ TLR4 inhibition increased the number of podocytes and played a role in improving renal inflammation and renal fibrosis.^182^, ^183^ JAK2/STAT3 was associated with podocyte hypertrophy.^96^ IRS1/PI3K/AKT signaling pathway was involved in glomerular podocyte EMT and glomerulosclerosis.^123^ Activating PI3K/AKT pathway could inhibit the Hippo pathway and led to nuclear YAP accumulation, thus accelerated glomerular mesangial cell proliferation in DN.^191^ Therefore, inflammation will lead to damage of the glomerular filtration barrier, thus resulting in glomerulosclerosis and proteinuria, which seriously affect the function of the kidney.

### 4.3 Retinopathy

DR encompasses a spectrum of eye conditions that arise from microvascular damage to the retina due to diabetes. It is a chronic and progressive disease that can compromise vision and, in severe cases, lead to blindness. The precise mechanisms underlying DR remain incompletely understood, although a considerable body of research suggests a strong association with inflammation exacerbated by prolonged hyperglycemia.

#### 4.3.1 Inflammatory damage to retinal structures

Inflammation seriously affects the structural changes of the retina. Excessive inflammatory response changes vascular permeability, capillary occlusion, and angiogenesis, resulting in damage to the blood–retinal barrier and retinal fibrosis. Based on the progress of disease, DR is divided into nonproliferative and proliferative. The loss of pericytes in capillaries was one of the early pathological changes of DR.^192^ Due to loss of pericytes and the migration of endothelial cells, nonfunctional blood vessels appeared in the retina, and formed an ischemic and anoxic retinal environment, which promoted the formation of neovascularization and eventually developed into proliferative DR.^193^ In the condition of high glucose, IL‐1β induced apoptosis of pericyte cells and increased vascular permeability through NF‐κB signal pathway, and it could also stimulates TLR4 through AGE to increase angiogenic factor Galectin‐1 expression of macrophages and microglia.^194^ TNF‐α could affect the integrity of blood–retinal barrier in DM mice^38^ and this process was regulated by IL‐6.^61^ IL‐17A could damage retinal microvessels, retinal Müller cells, and ganglion cells.^63^ TGF‐β1 exerted a certain role in endothelial cell interaction and vascular remodeling.^195^ Adhesion molecule Ninjurin1 played an important role in maintaining vascular integrity mediated by macrophages.^196^ NLRP3 participated in the immune response of the retina and the pathological neovascularization in the late stage of DR.^197^ NLRP3‐caspase‐1‐GSDMD‐mediated pyrolysis might be the factor for the partial loss of retinal pericytes upon high glucose environment.^198^ CXCL1 could change the blood–optic retina barrier of DR through neutrophil recruitment.^100^ The activation of NF‐κB/STAT3 signal pathway could stimulate the polarization of M1 phenotypic microglia/macrophages and upregulated IL‐1β, IL‐6, and TNF‐α expression to demolish the retinal structure.^199^ The inhibition of JNK mitigated the pathological damage of retina and reduced apoptosis, thus improving DR.^200^ PI3K/AKT/STAT3/NF‐κB signal pathway could inactivate microglia and maintain the integrity of the blood–retinal barrier.^201^ Therefore, a variety of inflammatory cytokines, chemokines, inflammasome, and related inflammatory signaling pathway are involved in the structural changes of the retina especially blood–retinal barrier.

#### 4.3.2 Visual impairment and risk of blindness

**FIGURE 5:**
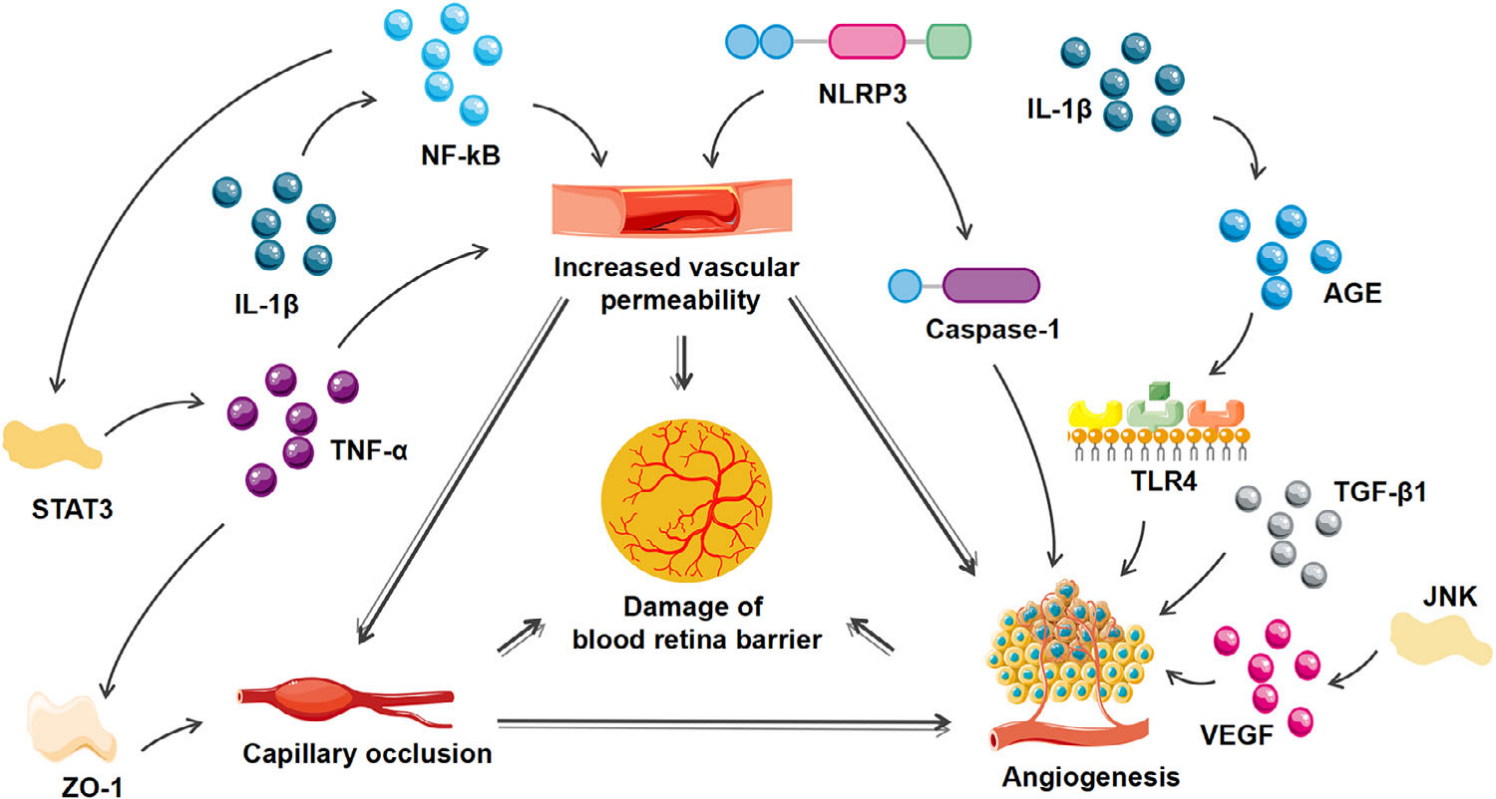
Inflammation damage to blood retina barrier. NLRP3 affects angiogenesis and capillary permeability by secreting proinflammatory cytokines. JNK activation can increase retinal VEGF expression and then speed up pathological retinal neovascularization. STAT3 activation increases TNF‐α expression and promotes ZO‐1 disintegration to damage blood–retinal barrier. IL‐1β increases vascular permeability through NF‐κB signal pathway, and it can also stimulate TLR4 through AGE to regulate angiogenesis.

During the progression of the disease, patients with DR would deteriorate from limited field of vision to blindness due to gradual destruction of the blood retina barrier and the increase of neovascularization.^202^ With sustained high glucose stimulation, the capillaries were destructed and their permeability was altered, and macular edema was formed subsequently, which lead to blurred or impaired vision. In addition, neovascularization in ischemic retina was prone to rupture, allowing blood to enter the eye, and further exacerbating the sudden loss of vision. Various inflammatory responses and signaling pathways were involved in vascular permeability and angiogenesis of DR. NF‐κB promoted the permeability of endothelial cell.^67^ O‐GlcNAcylation of NF‐κB increased the death of retinal ganglion cells in DR.^203^ TLR4/AGE pathway was activated by IL‐1β and then increased angiogenic factor Galectin‐1 of macrophages or microglia to promote angiogenesis in retina.^194^ JNK activation could increase retinal VEGF expression and then speed up pathological retinal neovascularization.^204^ STAT3 activation increased TNF‐α expression and promoted ZO‐1 disintegration to injure blood–retinal barrier.^61^ The activation of PI3K/AKT could mediate endothelial cells autophagy,^205^ slow down vascular endothelial cell–mesenchymal transformation,^206^ and improve visual impairment in DR. Therefore, inflammation aggravates the damage of blood–retinal barrier in DR patients and increases the risk of visual impairment and blindness (Figure 5).

### 4.4 Neuropathy

Diabetic neuropathy is among the most prevalent chronic complications of diabetes, affecting both the central and peripheral nervous systems. DPN is the most prevalent form of diabetic neuropathy, characterized by symptoms such as symmetrical pain and sensory disturbances that arise due to peripheral nerve dysfunction. This condition is known to be modulated by inflammation present in patients with DM.

#### 4.4.1 Inflammatory damage to nerve tissue

**FIGURE 6:**
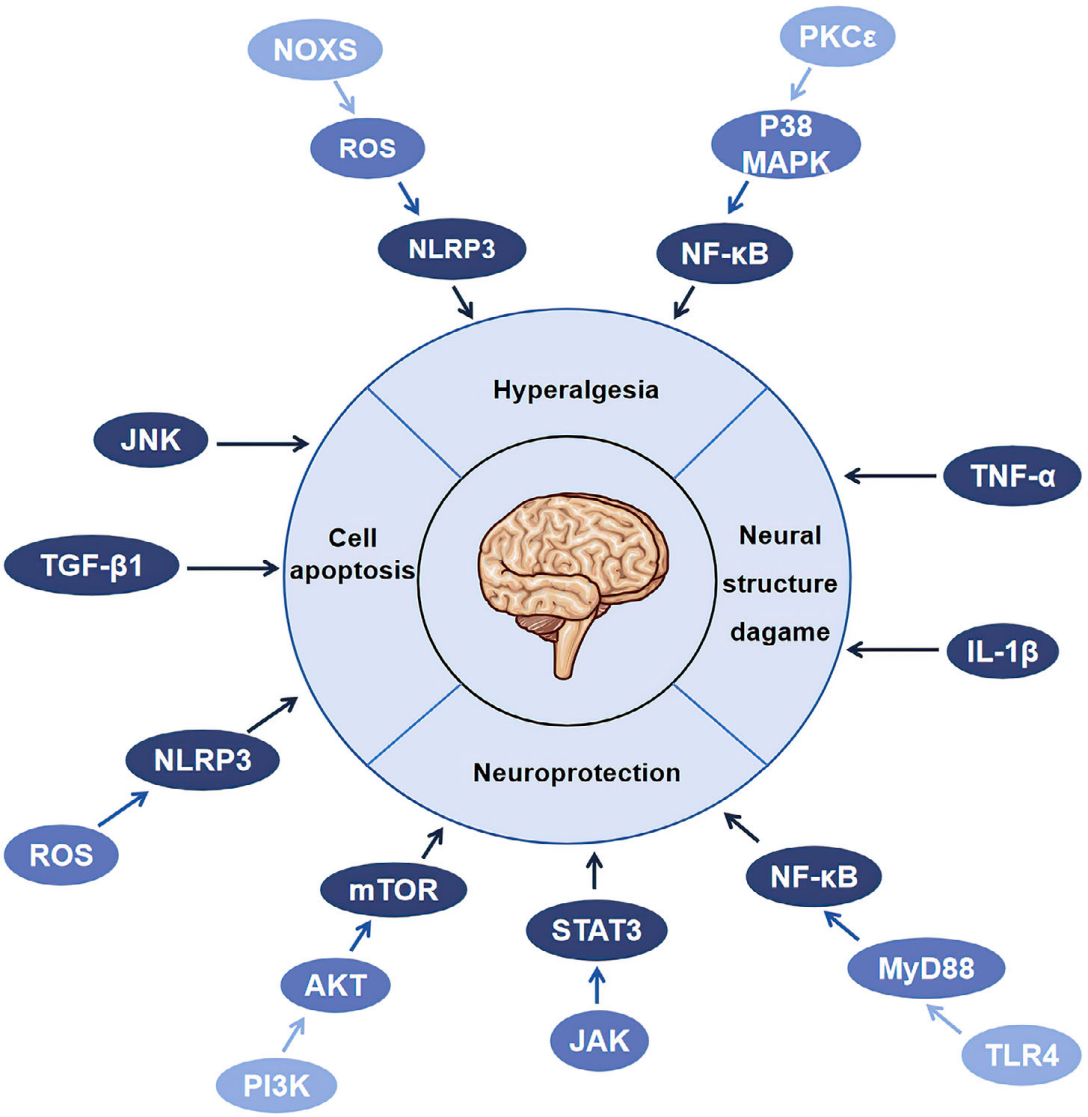
Inflammation damage to nerve tissue. Inflammatory reaction promotes the development of DPN, accompanied by cell apoptosis, neural structure damage, neuroprotection, and hyperalgesia. JNK, TGF‐β, and NLRP3 promote cell apoptosis. TNF‐α and IL‐1β damage nerve structure. TLR4/MyD88/NF‐κB, JAK/STAT3, and PI3K/AKT/mTOR exert a neuroprotective effect. PKCε/P38MAPK/NF‐κB and NOXS/ROS/NLRP3 aggravate hyperalgesia.

Inflammatory cytokines, immune cells and inflammasome participated in regulation of nerve conduction velocity, nerve regeneration function, neuronal apoptosis, neurovascular function and hyperalgesia (Figure 6). TNF‐α slowed down the nerve conduction velocity and disrupted the lamellar and axonal structures.^41^, ^42^, ^43^ IL‐1β and its receptor damaged the ultrastructure and axon of myelin in DPN.^73^ TGF‐β triggered neuronal apoptosis by inducing inflammation.^87^ NLRP3 was activated by excessive ROS and led to pyroptosis of Schwann cells.^207^ Adhesion molecules might slow down the motor nerve conduction velocity in DPN.^208^ Related inflammatory signal pathways also affected the structure and function of nerve tissue. Activation of NF‐κB by targeting genes of miR‐146 was related with inflammation, peripheral tissue blood perfusion, and thrombosis in DPN.^209^ Ciliary neurotrophic factor could promote axons regeneration and protect the peripheral nerve by activating NF‐κB.^210^ TLRs could stimulate the secretion of cytokines and chemokines,^211^, ^212^ affect the early neuropathy of sensory neurons through immunomodulation or recruitment,^211^ and regulate axonal growth and apoptosis of dorsal root ganglion neurons under hyperglycemia.^213^ TLR4/MyD88/NF‐κB signal pathway exerted a neuroprotective effect,^214^ while JNK,^215^ JAK/STAT3, and PI3K/AKT/mTOR pathway^216^, ^217^ were involved in the apoptosis, autophagy, and proliferation of Schwann cells. Therefore, inflammation can damage nerve tissue through various pathways and aggravate the progress of DPN.

#### 4.4.2 Sensory and motor nerve dysfunction

Loss of sensory function was one of the early manifestations of diabetic neuropathy, which was characterized by the acquisition or loss of sensory function.^218^ In the late stage of the disease, patients with DN appeared neuromuscular damage, resulting in motor disorders, and impacted on daily life.^219^ Similar to neuropathic pain‐like symptoms, the loss of sensory function was related by inflammatory. NLRP3 was activated in dorsal root ganglion, which could increase mechanical hyperalgesia in DPN.^220^ Moreover, activating NLRP3 in the peripheral nerve caused paclitaxel‐induced neuropathic pain.^221^ PKCε/P38MAPK/NF‐κB and NOXS/ROS/NLRP3 were involved in inflammatory of hyperalgesia.^222^, ^223^ The chemokine SDF‐1 and its receptor CXCR4 could mediate calcium influx and excitability of dorsal root neurons, which lead to neuropathic pain.^104^ Additionally, inhibition of T cell infiltration was of positive significance in improving peripheral neuropathic pain in DPN.^224^ Therefore, all kinds of inflammatory reactions will aggravate the sensory and motor dysfunction in patients with DN.

Considered collectively, excessive inflammation can intensify the progression of diabetes‐related complications, resulting in organ damage, including the heart, kidneys, brain, and eyes. A comprehensive investigation into the potential targets within the inflammatory process may represent one of the strategic approaches for the treatment and prevention of these diabetes‐related complications.

## 5 THERAPEUTIC INTERVENTIONS TARGETING INFLAMMATION IN DIABETES

As inflammation exists throughout the pathological process of DM, it has become one of the important targets for the treatment of DM. At present, anti‐inflammatory drugs and lifestyle improvement are commonly used in clinical treatment of DM.

### 5.1 Anti‐inflammatory drugs

Nonsteroidal anti‐inflammatory drugs (NSAIDs), corticosteroids and immunosuppressive agents are anti‐inflammatory drugs commonly used in the treatment of DM. NSAIDs could regulate blood glucose and improve insulin sensitivity by anti‐inflammatory effect.^225^ It reduced the incidence of cardiovascular events in patients with DM, while might increase the risk of bleeding to some extent.^226^ For example, salsalate, one of NSAIDs, could reduce inflammatory parameters such as CRP, free fatty acid in obese individuals at high risk of DM.^225^ Anti‐inflammatory effect of salsalate could improve blood glucose parameters and insulin sensitivity.^227^ However, it might be not beneficial to cardiac and renal function.^228^ Indomethacin could not only stimulate the production of endogenous glucose in T2DM patients by inhibiting insulin secretion,^229^ but also reduced the increase of albumin excretion rate in T1DM patients with microalbuminuria by inhibiting renal prostaglandin synthesis.^230^ Additionally, indomethacin could also improve the cerebrovascular reactivity in patients with DM.^231^ Celecoxib, as a cyclo‐oxygenase‐2 inhibitor, had a certain efficacy in the treatment of early DR and it could play an anti‐inflammatory role in reducing fluorescein leakage.^232^ It was worth noting that indomethacin and celecoxib might damage renal function.^231^, ^232^ Diclofenac could inhibit postoperative inflammation and control intraocular pressure in patients with diabetic macular edema, but its inhibitory effect decreased with the extension of operation time.^233^ At the same time, studies found that diclofenac could be used as an effective analgesic before treatment, reducing the pain during surgical treatment of DR,^234^ and preventing early macular thickening after cataract surgery in patients with nonproliferative and mild nonproliferative DR.^235^ Diclofenac should be used with caution in the treatment of DM patients with heart disease because it will aggravate cardiovascular disease.^234^ Curcumin could reduce the average concentration of high‐sensitivity CRP in DM patients, suggesting it reduced the risk of diabetes complications by reducing inflammatory reaction.^236^ Further research found that in patients who met the criteria for prediabetes and were treated with curcumin, the levels of the anti‐inflammatory cytokine adiponectin gradually increased, which could effectively prevent DM in prediabetic people.^237^ In addition, during the 9‐month treatment period, there were no significant adverse reactions in the curcumin treatment group, and only some subjects experienced mild symptoms such as itching, constipation, and dizziness.^237^

Corticosteroids and immunosuppressive agents regulated the number and activity of immune cells^238^, ^239^ as well as inhibited the activation and secretion of inflammatory cytokines and inflammasome.^240^ It was reported that rituximab could temporarily stabilize β‐cell function, trigger increases in T cell genes and decreases in B cell genes by analyzing the blood of patients with T1DM after rituximab treatment.^241^ It was suggested that the combination of rituximab and other drugs with the function of blocking T cell activity had a more longstanding clinical effect. However, a study of rituximab in the treatment of T1DM found that although rituximab delayed the progression of the disease, it increased the frequency of asymptomatic viremias caused by polyomavirus.^242^ The level of fibroblast growth factor‐21 (FGF 21) was closely related to inflammation and IR.^243^ Studies have found that FGF 21 levels in patients with DM decreased after treatment with dexamethasone, but the effect of its persistence needed more in‐depth study.^244^ What is more, dexamethasone could improve vision loss caused by macular edema.^245^ However, it caused a mild increase in glucose levels.^246^ Betamethasone could not only improve peripheral insulin sensitivity, but also inhibit systemic inflammation and control intraocular pressure in patients with diabetic macular edema,^233^, ^247^ while it might have a negative effect on bone formation.^247^ Azathioprine could improve β‐cell function, decrease glycosylated hemoglobin level and patients’ dependence on insulin therapy, and inhibit T‐cell proliferation, though it might cause vomiting and mild hair loss.^248^, ^249^ Abatacept improved immune cell subsets and insulin secretion in patients with T1DM, including reducing the frequency of inducible T‐cell costimulatory (ICOS)+ PD1+ Tfh cells, naive CD4+ T cells, and the frequency of CD4+ regulatory T cells (Tregs) upregualtion.^250^, ^251^ However, abatacept might induce skin and connective tissue disease in patients with DM.^250^ A random clinical trial of otelixizumab in the treatment of T1DM reported that the reactivation rate of Epstein–Barr virus (EBV) was associated with increased productive T cell clonality. It was found that otelixizumab could temporarily damage immune activity and allow EBV to be replenished in a dose‐dependence.^238^ In addition, otelixizumab was a chimeric CD3 antibody whose structure limited the ability to bind complement or Fc receptors, thereby reducing the risk of adverse clinical reactions caused by cytokine release.^239^ However, in clinical trials, participants taking otelixizumab got more than one adverse event due to cytokine dysfunction and the frequency and severity of adverse events were dose dependent.^238^ Teplizumab, an FcR non‐binding anti‐CD3 mAb, has also effective in the clinical treatment of diabetes. The study found that compared with the control group, immune activation‐related genes decreased, while genes expressions related to T cell differentiation and regulation increased in DM patients after treated with Teplizumab.^252^ Other study found Teplizumab reduced the circulating pool of CD3‐expressing lymphocytes, increased the proportion of CD8+ central memory T cells, effector cells, and programmed cell death protein 1+ (PD‐1+) cells, and regulated CD127 expression in circulating CD8 T cell subsets.^252^, ^253^ Teplizumab was also reported to preserve the function of β‐cells, but it might lead to adverse events such as headache, gastrointestinal symptoms, rash, lymphopenia, and mild cytokine release syndrome.^254^ Cilostazol could inhibit the activation of NLRP3 inflammasome in endothelial cells, thereby inhibiting the production of IL‐1β and IL‐18, suggesting it could reduce adverse vascular reactions and treat endothelial dysfunction in DM patients.^240^ While a case report showed that cilostazol could cause nephrotoxicity at any time after ingestion.^255^ Rapamycin affected DM patients directly by refitting the suppressive activity of CD4+ Tregs, though it did not directly alter effector T cell function.^256^ In a clinical trial, it was found that although rapamycin treatment of T1DM patients increased Tregs in the first month, it could also lead to transient β‐cell dysfunction.^257^ The common clinical drugs of anti‐inflammatory for DM were listed in Table 1.

**TABLE 1:** The anti‐inflammatory drugs used in diabetes.

Classification	Drugs	Function	Pharmacological mechanism	Side effect	References
Nonsteroidal anti‐inflammatory drugs (NSAIDs)	Aspirin	Anti‐inflammation Antiplatelet aggregation	Inhibiting cyclooxygenase activity	Bleeding risk increase	^226^, ^258^, ^259^, ^260^
	Salsalate	Regulating blood sugar Improving insulin sensitivity Anti‐inflammation Reducing the risk of cardiovascular disease	Inhibiting the level of glycosylated hemoglobin and C‐reaction protein Decreasing the number of leukocytes, neutrophils and lymphocytes	Injury of cardiac and renal function	^225^, ^228^
	Indomethacin	Regulating blood sugar Improving cerebrovascular reactivity Reducing proteinuria	Inhibiting insulin secretion Stimulating endogenous glucose production Reducing cyclooxygenase production and renal prostaglandin synthesis	Renal function damage	^229^, ^230^, ^231^
	Celecoxib	Anti‐inflammation Inhibition fluorescein leakage	Inhibiting cyclooxygenase activity Reducing VEGF expression	Renal function damage	^232^
	Diclofenac	Preventing intraocular pressure and cystoid macular edema Analgesia Anti‐inflammation	Inhibiting COX expression Blocking prostaglandin synthesis	Cardiovascular risk	^233^, ^234^, ^235^
	Curcumin	Anti‐inflammation Improving overall function of β‐cells	Reducing C‐reaction proteins Increasing anti‐inflammatory cytokines	Itching Constipation Vertigo	^236^, ^237^
Corticosteroids and immunosuppressive agents	Rituximab	Anti‐inflammation Regulating blood sugar Stabilize β‐cell function	Increasing heterogeneous T cell population Inhibiting B cell activity	Increasing the frequency of asymptomatic viremias	^241^, ^242^
	Dexamethasone	Protecting against cystoid macular edema Anti‐inflammation	Decreasing fibroblast growth factor	Mild increase in glucose	^244^, ^245^, ^246^
	Betamethasone	Regulating blood sugar Anti‐inflammation	Reducing endogenous glucose production Regulating cyclooxygenase expression	Affecting bone formation	^233^, ^247^
	Azathioprine	Regulating blood sugar Improving insulin resistance	Decreasing glycosylated hemoglobin level Affecting T‐cell proliferation	Vomiting Mild hair loss	^248^, ^249^
	Abatacept	Regulating immune cell subsets Affecting insulin secretion	Reducing CD4 central memory T‐cell and CD4+ regulatory T cells Increasing naive CD4+ T cells	Inducing skin and connective tissue disorders	^250^, ^251^
	Otelixizumab	Regulating immune cell subsets Anti‐inflammation	Inhibiting T cells Reducing cytokines level	Headache Nausea Vomiting Rash	^238^, ^239^
	Teplizumab	Regulating immune cell subsets Anti‐inflammation	Increasing proportions of CD8+ central memory T cells, effector memory and PD‐1+ cells Reducing CD4+ effector memory T cells	Headache Gastrointestinal symptoms Rash Lymphopenia Mild cytokine release syndrome	^252^, ^253^, ^254^
	Cilostazol	Anti‐inflammation Reducing endothelial dysfunction	Reducing the activity of NLRP3 inflammasome, IL‐1β and IL‐18 Increasing sirtuin 1	Inducing nephrotoxicity	^240^, ^255^
	Rapamycin	Anti‐inflammation Reducing insulin requirement	Refitting CD4+ regulatory T‐cells function	Inducing transient β‐cell dysfunction	^256^, ^257^

### 5.2 Lifestyle modifications

#### 5.2.1 Healthy diet and exercise

It has been reported that exercise could control blood sugar, increase insulin sensitivity, lose weight, reduce cardiovascular risk factors, and improve life well‐being in diabetes.^261^, ^262^ Sedentariness should be interrupted every 30 min, which was benefit to adults with T2DM. Besides, regular physical exercise was of great benefit to patients with DM, because it in part could downregulate pro‐inflammatory cytokines, inhibit inflammasome, and upregulate anti‐inflammatory cytokines. The study found that high sensitivity CRP and IL‐18 decreased and IL‐10 increased in DM patients in the exercise group compared with the control group.^263^ 12 weeks of Tai chi training^264^ or 6 months of exercise^265^ could decrease the activity of NLRP3 inflammasome and related inflammatory cytokines like IL‐1β and IL‐18 in prediabetic patients to alleviate systemic inflammation, reduce blood glucose, and improve IR. A single dose of high‐intensity interval training (HIIT) had obvious anti‐inflammatory effects on patients with T2DM, which was characterized by the decrease of TNF‐α and TLR2 surface protein.^266^ Except for HIIT, moderate continuous training (MCT) also played a positive role in patients with DM. It was found that both HIIT and MCT owned favorable adaptability to IL‐6, but only HIIT group could improve the blood lipid status of DM patients.^267^ Previous data showed endurance training significantly decreased the gene expression of NLRP3, P38MAPK, TNF‐α, and IL‐1β in the spinal cord of DM rats.^268^ Therefore, exercise played a pivotal role in improving the level of NLRP3 inflammasome and its related inflammatory factors. In future, it is worth to study the effects of exercise type, intensity and duration on DM patients. At the same time, it is necessary to make comprehensive use of exercise, drug therapy, and diet management to find out a better individual treatment plan.

Studies have shown that about 70% of global T2DM was caused by poor diet due to insufficient intake of whole grains and excessive intake of refined grains and processed meat.^269^ Therefore, a healthy and regular diet was particularly crucial for patients with DM. In a short‐term study, vitamin C was found to improve blood glucose control and blood pressure in patients with T2DM.^270^ Supplementation of vitamin D in patients with abnormal glucose homeostasis could reduce the expression of related inflammatory cytokines, such as CRP, TNF‐α, and IL‐6.^271^ A healthy diet could improve inflammation in overweight or obese people and reduce risk of developing DM.^272^ As compared with traditional low‐fat diet, traditional low‐carbohydrate diet could regulate blood glucose and downregulate inflammatory cytokines, suggesting low‐carbohydrate diet could improve the subclinical inflammatory state of T2DM patients.^273^ Whole‐grain diet could reduce body weight, serum inflammatory markers, IL‐6, CRP, to improve low‐grade systemic inflammation, even though it did not change insulin sensitivity.^274^ As compared with the control group, almonds decreased the level of IL‐6, CRP, and TNF‐α in T2DM patients.^275^ Other study also found that nut intake was negatively correlated with inflammation and markers of blood glucose/insulin homeostasis.^276^ Therefore, it is helpful to reduce the intake of harmful dietary diseases and eat protective diets appropriately, such as, yogurt, fruits, whole grains, nonstarchy vegetables, nuts, and seeds.

#### 5.2.2 Weight management and inflammation reduction

The inflammatory reaction could induce abnormal insulin secretion of β cells, which was one of the characteristics of T2DM.^277^ Studies have found that obesity was a strong driving force for the occurrence and development of DM, might affect the early β‐cell function and cell fate.^278^, ^279^ Meanwhile, when DM patients were overweight or obese, excessive adipose tissue would release related inflammatory mediators to facilitate the development of DM. Multiple inflammatory signaling pathways were closely related to the occurrence of obesity, such as MAPK, PI3K, JAK/STAT, JNK, which were also essential to DM.^280^

Taken together, anti‐inflammatory drugs and good living habits had positive significance for DM patients. However, the current clinical use of drugs targeting inflammation might cause a series of side effects in patients, and its development still needs a large number of in‐depth clinical trials. Moreover, not all patients with diabetes can use anti‐inflammatory drugs, and the appropriate treatment plan needs to be chosen according to the specific situation in clinical use. Of note, it also needs to pay attention to individual differences over the relationship between inflammation and diabetes to achieve more accurate and effective treatment strategies.

## 6 CONCLUSION AND FUTURE DIRECTIONS

This review concentrates on the molecular mechanisms underpinning inflammation in diabetes complications, encompassing the activation of immune cells, interactions between cytokines and the signaling pathways involving IR and inflammation, and the consequences of inflammation on diabetes‐related complications. Furthermore, the article summarizes existing interventions that aim to mitigate diabetes complications through the modulation of inflammation.

Inflammation plays a pivotal role in the development and progression of complications in diabetes, a chronic condition. Targeting inflammation has emerged as a promising therapeutic approach for managing diabetes complications. For instance, the development of inhibitors that can regulate or control the activity of proinflammatory cytokines may help slow down the advancement of the disease. Similarly, designing drugs that can specifically modulate the balance of immune cell populations or prompt a shift in cell state could potentially lead to the recovery or improvement of diabetes‐related conditions.

However, the current researches on the relationship between inflammation and diabetes complications are not enough. Due to the complex inflammatory process in the pathogenesis of diabetes, there are still much more problems to be explored. For instance, it is not yet known whether inflammatory signaling pathways will change in line with the diabetes progress or whether the treatment of some inflammatory drugs will lead to new complications of diabetes, or if there are clear inflammatory markers to reflect the inflammatory progression of DM patients. Also, it is worth noting that there is no clear inflammation model that can simulate clinical diabetes, and a large number of clinical samples are still needed to determine the exact significance of inflammation indicators and the relationship between inflammation and diabetes. Although there are still many treatments of diabetes complications, unique drugs targeting inflammation and with fewer adverse reactions are still ready to be designed. Therefore, there needs to be more in‐depth study on the inflammatory process in diabetes complications by the combination of basic research and clinical practice. Of note, inflammation treatment strategies should be combined with other treatment methods, such as drug therapy, diet management, and exercise, to get better effects in future.

## AUTHOR CONTRIBUTIONS

*Writing—original draft*: Lu Zhao. *Visualization*: Haoran Hu. *Literature search*: Lin Zhang, Zheting Liu, Yunchao Huang, and Qian Liu. *Writing—reviewing*: Liang Jin. *Editing and supervision*: Meifei Zhu. *Conceptualization, writing—reviewing, editing and supervision*: Ling Zhang. All the authors have read and approved the final version of the manuscript.

## CONFLICT OF INTEREST STATEMENT

All authors have declared that no conflict of interest exists.

## ETHICS STATEMENT

Not applicable.

## Data Availability

Not applicable.
